# Introducing and Testing the Creepiness of Situation Scale (CRoSS)

**DOI:** 10.3389/fpsyg.2018.02220

**Published:** 2018-11-16

**Authors:** Markus Langer, Cornelius J. König

**Affiliations:** Fachrichtung Psychologie, Universität des Saarlandes, Saarbrücken, Germany

**Keywords:** scale development, creepiness of situations, reliability and validity, technology acceptance, uncanny valley

## Abstract

When people interact with novel technologies (e.g., robots, novel technological tools), the word “creepy” regularly pops up. We define creepy situations as eliciting uneasy feelings and involving ambiguity (e.g., on how the behave or how to judge the situation). A common metric for creepiness would help evaluating creepiness of situations and developing adequate interventions against creepiness. Following psychometrical guidelines, we developed the Creepiness of Situation Scale (CRoSS) across four studies with a total of *N* = 882 American and German participants. In Studies 1–3, participants watched a video of a creepy situation involving technology. Study 1 used exploratory factor analysis in an American sample and showed that creepiness consists of emotional creepiness and creepy ambiguity. In a German sample, Study 2 confirmed these subdimensions. Study 3 supported validity of the CRoSS as creepiness correlated positively with privacy concerns and computer anxiety, but negatively with controllability and transparency. Study 4 used the scale in a 2 (male vs. female experimenter) × 2 (male vs. female participant) × 2 (day vs. night) field study to demonstrate its usefulness for non-technological settings and its sensitivity to theory-based predictions. Results indicate that participants contacted by an experimenter at night-time reported higher feelings of creepiness. Overall, these studies suggest that the CRoSS is a psychometrically sound measure for research and practice.

## Introduction

Technology advances rapidly leading humans to be constantly confronted with novel and unknown situations. For instance, there are virtual characters instructing people how to adapt their non-verbal behavior in social situations (Langer et al., 2016), computer avatars are used for therapy of psychological disorders (e.g., schizophrenia; Craig et al., 2018), and algorithms decide who you could date next (Toma, 2015). Situations involving novel technologies often lead to ambiguous situations - situations that are hard to judge and in which people do not really know how to behave (Shklovski et al., 2014; Tene and Polonetsky, 2015). In these situations, people might have queasy feelings that are hard to describe or express (Tene and Polonetsky, 2015). Imagine, for example, situations in which virtual agents or robots become very human-like but there are still slight differences in behavior or appearance between them and humans. In such situations, people commonly report that they felt uneasy when introduced to virtual agents or robots – a phenomenon that is referred to as the uncanny valley (Mori, 1970; Mori et al., 2012).

For lack of a better description, people tend to refer to ambiguous situations, or ones they have difficulty judging, or that evoke uneasy feelings as “creepy.” Feelings of creepiness can also arise in interpersonal situations, for example when meeting strangers (McAndrew and Koehnke, 2016). However, research on creepiness is still in its infancy as it is not yet clear what constitutes creepiness and what are the antecedents and consequences. As such, a common metric for assessing the creepiness of situations could aid not only research regarding encounters with new technology and strangers but also has several practical uses. For instance, movie producers could evaluate the creepiness of their computer-animated movies and try to decrease such feelings before the release date, which could potentially help prevent movie flops (cf., Geller, 2008; Misselhorn, 2009). More generally speaking, creepiness would become comparable between situations and therefore better understandable, predictable, and preventable.

As of yet, no psychometrically sound measure of creepiness exists. Therefore, the aim of the current four studies was to develop and investigate the psychometric properties (i.e., dimensionality, reliability, convergent, and divergent validity) of the Creepiness of Situation Scale (CRoSS) to offer a consistent measure for creepiness that can be used for assessing the creepiness of everyday situations and novel technologies.

## Theoretical Background

### Creepiness

Creepiness is a rather new concept in research. In their study, ”On the nature of creepiness,” McAndrew and Koehnke (2016) analyzed creepy situations and why they were classified as such – for instance, why does being approached by a stranger in the night lead to feelings of creepiness (McAndrew and Koehnke, 2016). McAndrew and Koehnke (2016) argued that unpredictability evokes creepiness. For example, they proposed that people with unusual patterns of non-verbal behavior or physical characteristics outside the social norm (e.g., outstanding style of clothing) can elicit feelings of creepiness in other people as they seem to be less predictable than people who dress or behave more ordinarily. Thus, McAndrew and Koehnke (2016) argued that this unpredictability leads to uneasy feelings about these non-conformist people and to ambiguity about how to behave and how to judge them.

Another area of research offering particularly useful ideas to understanding creepiness is human-computer interaction. Within this field, scholars and practitioners (Seyama and Nagayama, 2007; Walters et al., 2008; Tinwell, 2009; Ho and MacDorman, 2010; Saygin et al., 2012; Kätsyri et al., 2015) extensively debate the phenomenon of the uncanny valley (Mori, 1970; Mori et al., 2012), which describes feelings toward robots or virtual agents. More precisely, the uncanny valley argument assumes that people accept virtual agents and robots more when they become more human-like but if their appearance becomes very human-like but they are still artificial in some way and people cannot point their finger to what makes the robots unhuman, acceptance drops rapidly (Kätsyri et al., 2015). The movie “Polar Express” is commonly cited as being a victim of the uncanny valley (cf., Geller, 2008; Walters et al., 2008; Ho and MacDorman, 2010; Kätsyri et al., 2015) as people tended to describe the characters within this movie as creepy, which is assumed to have impaired audience reactions toward the movie.

The drop of acceptance (i.e., the uncanny valley) is often assumed to be caused by feelings of creepiness when humans are exposed to robots or virtual characters (MacDorman, 2006; Mori et al., 2012; Kätsyri et al., 2015). The mismatch between the human-like appearance on the one hand, and the somewhat artificial behavior on the other hand might lead to a feeling of unpredictability of what the robot or virtual agent will be doing next (similarly to humans behaving strangely, see Kätsyri et al., 2015; MacDorman and Chattopadhyay, 2016). As a result, people feel uneasy about interacting with such robots and virtual agents, but they also feel ambiguity about how to behave and how to judge them.

Above and beyond creepiness in interpersonal situations and in human–computer interaction, Tene and Polonetsky (2015) provided an excellent overview of creepiness elicited by novel technologies and technologies used in novel situations. In their “Theory of Creepy,” Tene and Polonetsky include examples of technologies and situations involving the use of technology which are supposed to be creepy. For example, they describe personalized analytics (i.e., exploiting users’ information on social media or web searches for personalized advertising) as potentially creepy. An example of creepy personalized analytics are algorithms predicting whether there is a pregnant person in a household and when the person will give birth (see Tene and Polonetsky, 2015), thus personalized advertising for baby products is provided. This might be useful for organizations selling baby products, but people who are confronted with such personalized advertisements might feel uncomfortable because they do not really know why websites they visit are suddenly providing them with suggestions on where to buy baby products. This feeling might be produced by unpredictability about which information “the web” has gathered about them and by uncertainty about how this advertisement has been produced.

Another example of a creepy situation is a situation where social listening is applied. Tene and Polonetsky (2015) describe a situation where a person having problems with their TV calls their friend for help. Shortly after making the phone call, the person is contacted by the TV’s producing company offering help with the TV. However, the user has no idea how the company knew there was an issue with the TV. It could be that the TV producing company monitors all problems with their TVs and calls users having severe issues. It could also be that the user assumes that the company has monitored their call with their friend. The unpredictability of the companies’ behavior can lead to uneasy feelings and feelings of ambiguity on how to judge the situation (e.g., “is it good that they want to help me or is it bad because they listen in on all my phone calls?”) or how to behave during the situation (Shklovski et al., 2014; Tene and Polonetsky, 2015).

Considering all the aforementioned work on creepiness, creepiness seems to be elicited by unpredictable people, situations, or technologies, and it seems that this induces rather unclear feelings of discomfort paired with uncertainty about how to behave during a creepy situation or with a creepy person or technology. Therefore, we can define creepiness as a potentially negative and uncomfortable emotional response paired with perceptions of ambiguity toward a person, technology or even during a situation. Furthermore, we can preliminarily assume that creepiness consists of two subdimensions, emotional creepiness and creepy ambiguity, and preliminarily define emotional creepiness as a rather unpleasant affective impression elicited by unpredictable people, situations, or technologies and creepy ambiguity as a lack of clarity on how to act and how to judge in such a situation.

Previous studies capturing creepiness have not used a consistent creepiness scale, nor did they investigate the psychometrical properties of their creepiness measures. In all of these studies, creepiness was measured with a single item (e.g., *not at all creepy* to *very creepy*, see McAndrew and Koehnke, 2016, and also Inkpen and Sedlins, 2011; Watt et al., 2017). This might be useful to capture the general creepiness of a situation, but it makes it hard to determine reliability. Moreover, this kind of measure does not distinguish emotional parts of creepiness from the ambiguity parts. Therefore, it is harder to discern what exactly about the situation has led to high creepiness values. Distinguishing between emotional creepiness and creepy ambiguity might help to understand which part of a situation needs to be adjusted to decrease creepiness.

At this point it is necessary to differentiate between creepiness and eeriness (Ho and MacDorman, 2010; Burleigh et al., 2013). Although both creepiness and eeriness describe similar negative reactions to strangeness and unfamiliarity and both are associated with emotions such as disgust, shock, and nervousness (Ho et al., 2008), there are also differences between the way previous research has described eeriness and the way we have defined creepiness (see above). First, the term eeriness is very closely associated to research about the uncanny valley and therefore about reactions to robots or virtual characters (Kätsyri et al., 2015). Therefore, eeriness is nearly exclusively applied to research comparing different humanoid robots or versions of virtual characters, which is further supported considering that the eeriness scale developed by Ho and MacDorman (2010) seems to be tailored to such research (e.g., items such as “without a definite lifespan – mortal” might only be used in research on robots and virtual characters). In contrast, the concept of creepiness can be applied to a broader range of situations: It relates to situations where people find themselves in interpersonal situations (McAndrew and Koehnke, 2016) or to situations where people interact with novel technologies (Tene and Polonetsky, 2015). Second, although creepiness should consist of an emotional response similar to the emotional response within sensations of eeriness, there should also be a cognitive response to the ambiguity and unpredictability of the situation, which is not a part of the eeriness concept (Ho et al., 2008). Third, eeriness seems to be especially related to fear (Ho et al., 2008), whereas creepiness might rather correlate with anxiety, and fear and anxiety needs to be differentiated: Fear is usually directed to a threatening object or situation where it evokes flight or fight tendencies, whereas anxiety emerges in situations where there is an ambiguous, unclear threat eliciting tension and where people prepare their escape or a potentially upcoming fight (Öhmann, 2008). As a result, creepiness with its ambiguous nature, where people potentially cannot point the finger to what exactly bothers them in a certain situation (e.g., whilst interacting with novel technologies), should result in tension and a state of preparedness because something bad could happen during a creepy situation. Fourth, there is some initial evidence by Ho et al. (2008) who were able to measure eeriness and creepiness as two distinct constructs. Consequently, we argue that creepiness and eeriness are distinct in a way that creepiness may be applied to a broader range of situations and that creepiness should not only consists of an emotional but also of a more cognitive response to creepy situations.

In the following sections, we will describe the scale development approach for the CRoSS in which we closely followed recommendations by Hinkin (1998). The scale development process consisted of four studies. In the first study, we collected data from an American sample on our initial set of items to carry out an exploratory factor analysis (EFA) to enhance understanding of the dimensions of creepiness. Please note that Fabrigar et al. (1999) suggested that researchers should have an idea about the potential subdimensions resulting from an EFA before conducting it. Additionally, we reduced the amount of items to increase efficiency of the scale. In the second study, we collected data from a German sample to apply a confirmatory factor analysis (CFA) to support the factors found in the first study. In the third study, we examined the convergent (using privacy concerns, transparency, controllability and computer anxiety) and divergent validity (using extraversion and conscientiousness) of the CRoSS. In the last study, we used the CRoSS in a field experiment to provide further validity evidence, to show that it is sensitive to experimental manipulations based on theoretical assumptions, and to show that the CRoSS is useful in situations extending beyond the use of technology. In this study, experimenters (male vs. female experimenters) approached people on the street (male vs. female participants) to respond to a questionnaire, either during the day or at night.

### Item Generation

The authors consulted the literature for studies on creepiness to obtain an overview of existing theories and measurement models of creepiness. Based on the research of Mori (1970), Mori et al. (2012), Shklovski et al. (2014), and Tene and Polonetsky (2015), and McAndrew and Koehnke (2016), the authors discussed the definition of creepiness and the proposed dimensionality of creepiness. We developed 14 items (see Table 1), following the guidelines of Hinkin (1998) (e.g., short statements, one idea per item). These items were intended to capture the facets of creepiness as inferred by prior research (i.e., emotional creepiness and creepy ambiguity). The six items that should capture emotional creepiness were written to represent unclear and queasy feelings toward a situation, whereas the eight items for creepy ambiguity were written to reflect uncertainty on how to judge a situation and how to behave during a situation. Items were generated in German, translated to English, sent to a native English-speaking proofreader, translated to German and checked for coherence with the original items. Concerning the response format, we consulted research by Lozano et al. (2008). According to their findings, a seven-point rating scale should provide a good foundation for obtaining adequate psychometric properties of a newly developed scale. Therefore, we decided to use a seven-point rating scale from 1 (*Strongly Disagree)* to 7 (*Strongly Agree).*

**Table 1:** Initial items in German and English, proposed dimensions of these items, and results of the exploratory factor analysis.

Item	Original item in English	Original item in German	Rotated loadings(all items)		Rotated loadings(after item reduction)	
				
			Factor 1	Factor 2	Factor 1	Factor 2
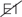	This was a strange situation.	Diese Situation war merkwürdig.	0.43	0.33	–	–
E2	During this situation, I had a queasy feeling.	Ich hatte ein mulmiges Gefühl während der Situation.	-0.20	0.82	0.10	0.89
E3	I had a feeling that there was something shady about this situation.	Ich hatte während der Situation das Gefühl, dass etwas faul ist.	0.26	0.66	-0.10	0.60
E4	I felt uneasy during this situation.	Ich fühlte mich unwohl während der Situation.	0.03	0.76	-0.10	0.83
E5	I had an indefinable fear during this situation.	Während der Situation hatte ich eine undefinierbare Angst.	0.03	0.75	-0.10	0.79
E6	This situation somehow felt threatening.	Die Situation fühlte sich irgendwie bedrohlich an.	0.01	0.87	0.05	0.87
A1	I did not know how to judge this situation.	Ich wusste nicht wie ich die Situation einschätzen sollte.	0.87	-0.14	-0.86	-0.17
A2	During this situation, I did not know exactly what was happening to me.	Ich wusste während der Situation nicht genau, was mit mir passiert.	0.76	0.12	-0.81	0.14
A3	During this situation, things were going on that I did not understand.	Während der Situation sind Dinge vorgegangen, die ich nicht verstanden habe.	0.77	0.07	-0.82	0.09
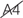	During this situation, I did not know if how I was being treated was OK.	Während der Situation wusste ich nicht, ob es in Ordnung ist, was gerade mit mir gemacht wird.	0.42	0.53	–	–
A5	I did not know exactly how to behave in this situation.	Ich wusste nicht genau, wie ich mich in dieser Situation verhalten sollte.	0.79	0.06	-0.84	0.07
A6	I did not know exactly what to expect of this situation.	Ich wusste nicht genau, was ich in der Situation zu erwarten habe.	0.52	0.00	-0.71	0.11
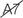	This situation was unpredictable.	Die Situation war unvorhersehbar.	0.26	0.19	–	–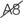	I had a feeling that I was not in control of the situation.	Ich hatte das Gefühl, keine Kontrolle über die Situation zu haben.	0.16	0.23	–	–


*E, emotional creepiness; A, creepy ambiguity, 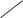, These items were removed from the scale after the exploratory factor analysis.*

## Study 1^1^: Exploratory Factor Analysis, And Scale Analysis In An American Sample

### Study 1: Method

Following Hinkin’s (1998) and Fabrigar et al. (1999) recommendation, we used EFA to examine the dimensionality of the scale, assessing factor loadings for the items, and potentially excluding items from the scale.

Amazons’ Mechanical Turk (MTurk; Buhrmester et al., 2011; Landers and Behrend, 2015) was used to collect data for the EFA. Following suggestions by Bortz and Schuster (2010) as well as Hinkin (1998) regarding required sample size for an EFA, we collected data until our final sample consisted of 300 participants (46% female) from the United States with a mean age of 36 years (*SD* = 10.98). The MTurk participants received a small amount of money for participating. For this study as well as for all of the following studies, participants were informed that they provide consent and agree that their data will be used for research purposes by continuing the respective study. During Study 1, participants watched a video where a situation similar to one of the creepy situations described by Tene and Polonetsky (2015) was shown. The video was recorded with a camera in the first person view to enhance participants’ immersion. In this video, a person sits in front of a computer screen using a word processing software when suddenly the computer produces an audible error signal; the person uses the mouse but nothing happens (i.e., the screen freezes). As a result, the person turns off the computer. Following, the person tries to restart the computer, but it does not turn on again. Afterward, the person reaches for their smartphone and starts texting a friend for help. In the video, the screen of the smartphone is visible so participants can read what the person is writing. It is also made clear that the person is writing to a friend, because there is already a texting history clarifying that they know each other (i.e., a message is visible from some hours ago; the person in the video addresses the friend with “buddy”). Once it is clear that the person asks a friend for help but before the message is sent to the friend, the person receives a call from an unknown number and starts acting confused over the call (e.g., hesitates to answer the call, uses confused hand gestures). The person presses the button to answer the call. Then the caller with a foreign accent starts speaking and says: “Hello? This is Chris from Computer Solutions. We heard that you are having problems with your computer? You were writing something but suddenly you could not move the mouse anymore and now the computer is not turning on again? Fortunately, this is a common problem with your computer series, I can help you fix this right now. You just need to execute the following steps…”. We dubbed this phone call to ensure that participants can hear it loud and clearly. Then, the video fades out without any further information. After watching the video, participants completed the 14 initial CRoSS items and provided demographic information. Additionally, participants had to describe what happened during the situation as a manipulation check. The manipulation check was to ensure whether participants had watched the video attentively and to explore if they perceived the situation displayed in the video as ambiguously as intended (e.g., if different participants came up with different explanations on what has happened during the situation).

### Study 1: Results

As a first step of Study 1, we analyzed the open-ended manipulation check question that asked participants for a description of what has happened during the situation. Table 2 shows the most common explanations that participants came up with. These explanations showed that participants watched the video and that the video generated a variety of ideas about what has happened during the situation. These commentaries showed that the video evoked reactions varying from a neutral description of the situation to the fear of privacy invasion and a hacker attack. Furthermore, there was a substantial number of participants who described the situation as “creepy.”

**Table 2:** Explanation that participants came up with in Study 1.

Explanation type	Example
Creepy situation	–A girl was typing on her computer. Her mouse stopped working. She turned the computer off. Then she couldn’t get anything to work. She was texting her friend when all of a sudden somebody called her who knew what was going on with her computer. Creepy.
	–Person’s computer froze up and they didn’t know what to do so they turned off the computer and texted their friend for help, and almost instantly got a really creepy unsolicited call offering to help which was either some new terrible business idea or someone scamming the computer user.
	–Somehow the man who called saw my message and chimed in to help fix my computer problem, but this seems like a disturbing breech of privacy to me.
Hacker attack	–He was being scammed remotely. They shut down and locked his PC, then called him offering to help fix it.
	–Someone was able to take over the pc and make it stop working. Then they called – they’re going to ask for credit card info, etc. as they’re hackers and crooks trying to get me to give them personal info in order to steal it and use it.
	–He was a victim of some sort of Malware and basically his PC is now being held for a ransom. I’m a PC technician and I’ve seen this a lot come through my door
Users’ fault	–Dude’s mouse and keyboard stopped working so he shut off his computer which is the stupidest first move anyone could do in that situation.
	–Her computer froze up and she SERIOUSLY didn’t even bother to ctrl+alt+del to see if it was the program malfunctioning and instead went RIGHT for the shutdown like some kind of noob.
	–Guy was trying to get his homework done. He unplugged the mouse and claimed to be having trouble with it. He made an excuse up to not to the work.
Description of the situation	–A lady was typing something and her computer’s mouse stopped working. She turned off the computer and was called by customer support.
	–A person was typing and the computer froze. Someone called saying they could help even though nobody was told about the trouble yet.
	–The man was working on his computer when it locked up on him and he turned it off. As he was texting a friend for help, his phone rang with a private number, and the person (with a foreign accent) on the other end was telling him that he was from computer support, that he could help him, if he fulfilled the following steps.

Based on the theoretical assumptions (Tene and Polonetsky, 2015; McAndrew and Koehnke, 2016), the two proposed scales of creepiness should be non-orthogonal (i.e., situations eliciting more ambiguity should also evoke more uneasy feelings). For the EFA we therefore used a principal component analysis with oblique rotation on the 14 CRoSS items. We chose oblique rotation in order to allow the two proposed scales of creepiness to be correlated (Fabrigar et al., 1999). To assess dimensionality, we used three criteria: The Kaiser-Guttman criterion (i.e., eigenvalues larger than 1; Kaiser, 1960), drops of eigenvalues in the scree plot, and comparison of the eigenvalues to random eigenvalues for 14 items with 300 participants (i.e., parallel analysis, Horn, 1965). Results indicate a two-factorial solution accounting for 61 percent of variance. We then analyzed the items regarding potential item removal (Hinkin, 1998). Ten of the initial fourteen items loaded substantially (>0.50) on their supposed factors, two items loaded on both factors equally (E1, A8) and two more items did not load substantially on any factors (A1, A5); accordingly, these four items were removed from the scale (cf., Hinkin, 1998) (see Table 1).

For the remaining ten items, we conducted another principal component analysis with oblique rotation that resulted in two factors explaining 68 percent of variance. Every item loaded substantially (>0.50) on its supposed factor. The correlation between the two factors was *r* = 0.52. In line with our initial idea about the potential dimensionality of creepiness, results showed a two-factor solution with five items on each factor. The first factor reflected emotional creepiness, with the items capturing an emotional response to a potentially creepy situation. The second factor reflected creepy ambiguity, with items describing insecurity about how to behave during the situation and how to judge the situation.

Furthermore, we conducted a scale reliability analysis to ensure reliability of the entire scale and the two subscales. For the entire scale we found a good reliability (cf., Cortina, 1993) of Cronbach’s α = 0.90 (emotional creepiness Cronbach’s α = 0.87; creepy ambiguity Cronbach’s α = 0.89).

## Study 2: Confirmatory Factor Analysis in a German Sample

### Study 2: Method

For the next step of the scale development, the goodness of fit of the resulting factor structure needs to be assessed (Hinkin, 1998). As such, we followed suggestions by Hinkin (1998) regarding the required sample size for a CFA and collected data from 306 German participants in an online study. Participants were recruited through social media, in psychology and economics courses at a German university, and on an online survey platform on which researchers take part in online surveys in exchange for other people to take part in their surveys. Three participants were excluded because of technical problems, and one participant was excluded because he stated that he did not take the study seriously. The final sample for Study 2 consisted of 302 German participants (67 percent female) with a mean age of 26 years (*SD* = 8.37). During the study, participants watched the same video as in the first study and afterward responded to the ten CRoSS items and to demographic questions. Similar to Study 1, participants had to describe what happened during the situation as a manipulation check.

### Study 2: Results

Similar to Study 1, we analyzed the open-ended manipulation check question; Table 3 shows the most common explanations that participants came up with. The only difference between the two samples was that no participant in the German sample questioned the abilities of the user in the video. Comparable to the American participants, the German participants explained the situation either very descriptively as it was, thought it was a “strange” situation or they imagined a hacker attack. This shows support for the fact that the situation in the video was also perceived ambiguously by the German participants.

**Table 3:** Explanation that participants came up with in Study 2.

Explanation type	Example
Creepy situation	–Problem with the computer. Suddenly a shady call. The caller inexplicably knows the problem and offers help.
	–The person has problems with the computer and texts a friend. Suddenly someone calls and says what the person texted the friend. This is totally crazy, like being under surveillance!
	–The moment the person who was writing on her computer wanted to contact a friend for help via smartphone, there was a call from customer support which strangely knew exactly what kind of a problem there was with the computer. Big brother is watching.
Hacker attack	–The computer was hacked and knocked out with a virus.
	–PC crashed during an important paper work. Maybe the virus reacted exactly to this situation and afterwards panic, fear, and helplessness of the user will be exploited. Wouldn’t happen to me as I work with cloud storage.
	–PC crash – restart fails – Whatsapp message to a friend – call from an unknown number – somebody who is obviously no native English speaker knows what has happened; new form of PC/smartphone/cloud hacking with potential service in return, key word: blackmailing???
Description of the situation	–There were word problems. Without asking for it, the support called the user to help.
	–The computer crashed. Whilst texting a friend and describing what has happened, a person from customer support called and already knew about the situation, without being informed before.
	–The computer crashed and a supposed employee of the customer support called with an anonymous number and knew details that he actually could not know.

*These explanations were translated from German.*

**FIGURE 1:**
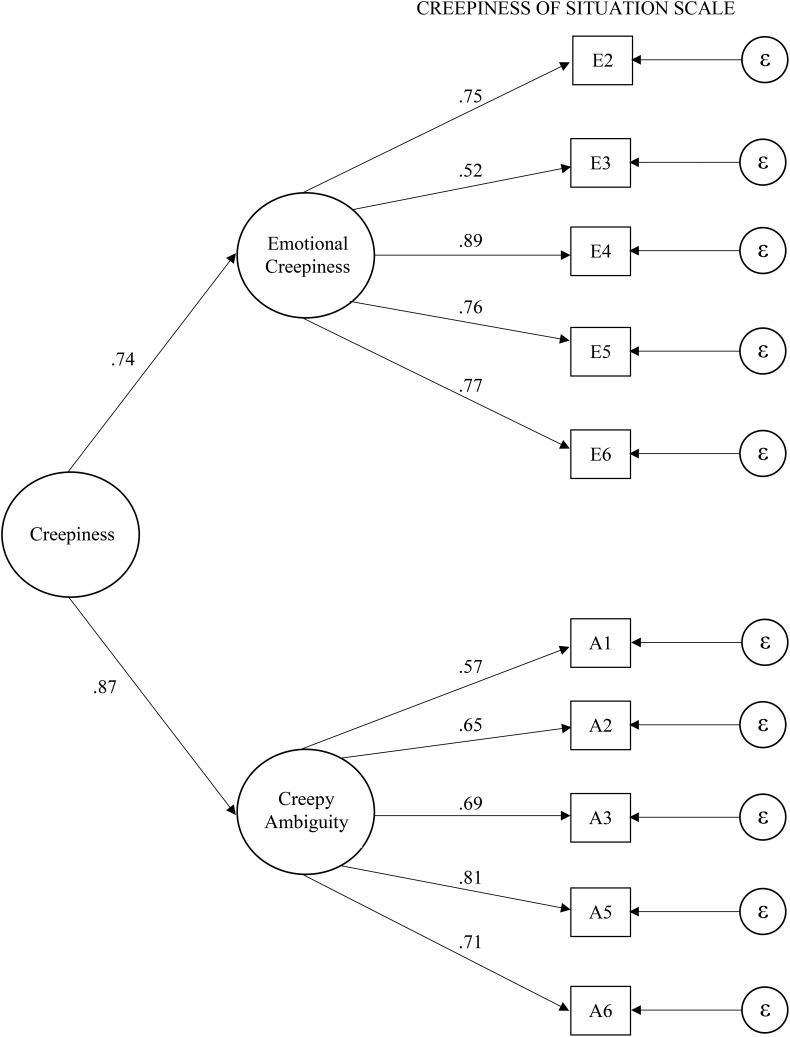
Resulting model of the confirmatory factor analysis in Study 2. Numbers represent standardized loadings. E2 – E6 = items of the scale emotional creepiness, A1 – A6 = items of the scale creepy ambiguity.

Since this sample was collected in Germany, where one might expect different results for the factors and reliability of the CRoSS compared to the American sample from Study 1, an EFA with oblique rotation was conducted for the items. Results showed two factors explaining 62 percent of the variance and all items loaded substantially (>0.50, see Figure 1) on their supposed dimension. The correlation between these two factors was *r* = 0.47. Furthermore, reliability for the scale was Cronbach’s α = 0.87 (emotional creepiness Cronbach’s α = 0.85; creepy ambiguity Cronbach’s α = 0.82). As the video in Study 1 was the same as in Study 2, we also compared creepiness ratings between the countries based on *N* = 602 participants (i.e., we combined the samples for this step of analysis). There were no significant differences between the countries for emotional creepiness (American *M* = 4.51, *SD* = 1.47; German *M* = 4.70, *SD* = 1.32, *t*[592.45] = 1.66, *p* = 0.10, *d* = 0.14), nor for creepy ambiguity (American *M* = 4.38, *SD* = 1.47; German *M* = 4.55, *SD* = 1.28, *t*[588.66] = 1.50, *p* = 0.13, *d* = 0.12). These results tentatively indicate that there are no substantial differences in the results of the EFA, the scale reliabilities, and the reactions to the video regarding creepiness of the American sample from Study 1 and of the German sample from Study 2.

Additionally, a CFA was conducted using the SPSS plugin AMOS. Creepiness consisted of the two factors emotional creepiness and creepy ambiguity, both loading on a common underlying factor called Creepiness. For this hypothesized model (Model 1 in Table 4, displayed in Figure 1), results showed that all of the paths between the factors and respective items were significant, as were the paths between the two factors and general creepiness. Furthermore, Table 4 shows fit indices of the proposed Model 1 in comparison to an alternative one-factor model and an orthogonal two-factor model. All things considered, Model 1 fits the data significantly better than the other two models regarding χ^2^ statistics, and it showed a better fit on all other fit indices. Although the χ^2^ statistic for Model 1 was significant, indicating a less-than-perfect fit for the proposed model, it should be kept in mind that the χ^2^ statistic is sensitive to sample size (Marsh et al., 1988); thus other fit indices should also be considered. They indicated an acceptable fit (root mean square error of approximation RMSEA = 0.08; MacCallum et al., 1996) or a good fit (for goodness-of-fit index GFI, adjusted goodness-of-fit index AGFI, >0.90 and comparative fit index CFI > 0.95; Bollen, 1990; Hu and Bentler, 1999).

**Table 4:** Model fit indices for the hypothesized Model 1 and two alternative models.

Model	χ^2^ (*df*)	Δχ^2^ (*df*)	CFI	GFI	AGFI	RMSEA
(1) Hypothesized correlated two-factor model	101.60^∗∗^ (34)	–	0.95	0.94	0.90	0.08
(2) One-factor model	310.04^∗∗^ (35)	208.44^∗∗^ (1)	0.79	0.78	0.65	0.16
(3) Orthogonal two-factor model	209.64^∗∗^ (35)	108.04^∗∗^ (1)	0.87	0.89	0.83	0.13


*Δχ^2^ indicates the difference between Model 1 and the respective model. CFI, Comparative Fit Index; GFI, Goodness-of-Fit Index; AGFI, Adjusted Goodness-of-Fit Index; RMSEA, Root Mean Square Error of Approximation.*

*^∗∗^*p* < 0.01.*

To conclude, the CFA indicated that the two factors emotional creepiness and creepy ambiguity which both loaded on the same general creepiness factor represented the data well. Accordingly, the next step of scale development is to gather evidence of construct validity. For this purpose, the next two sections cover an online and a field experiment to examine validity of the CRoSS.

## Study 3: Convergent and Divergent Validity

For the development of a new scale it is important to show that it is measuring a meaningful construct (Hinkin, 1998). Therefore, it is necessary to demonstrate correlations with other relevant constructs (convergent validity) and, at the same time, distinguishability from unrelated constructs (divergent validity). This scale development step is especially important in the case of creepiness, which research has just started to examine (cf., McAndrew and Koehnke, 2016).

### Convergent Validity

In an attempt to support convergent validity of the CRoSS, we propose correlations between creepiness and the constructs privacy concerns, computer anxiety, transparency, and controllability. Below, we provide theoretical support for each of the proposed correlations.

Privacy concerns are an important variable to measure feelings of privacy invasion through novel technologies (Smith et al., 2011). When people hold privacy concerns, they are under the impression that their personal data might be collected without their knowledge, that they have no control about which data are collected, that there might be errors in the data collection, and that personal data might be misused (Shin, 2010; Smith et al., 2011). Consequently, privacy concerns can lead to less trust in the organizations which elicited these concerns (Smith et al., 2011; Tene and Polonetsky, 2015). This can detrimentally affect important organizational outcomes such as, applicant reactions, provision of personal information, and online sales revenue (Phelps et al., 2000; Malhotra et al., 2004; Bauer et al., 2006; Shin, 2010).

Shklovski et al. (2014) proposed that creepiness will be present in situations where there are privacy concerns. For instance, they describe the invasion of privacy through smartphone apps. If people perceive privacy concerns because an app requests access to their pictures and contacts, although the app is for a game that has nothing to do with pictures or contacts, they can get a feeling that this somehow feels wrong (Shklovski et al., 2014). This feeling of “wrongness” (Shklovski et al., 2014, p. 2347) leads to users’ desire to distance themselves from the app to regain control over their privacy. As such, we propose that creepiness relates to privacy concerns as both feelings can be elicited through uncontrollable situations (see also Phelps et al., 2000; Shin, 2010). In fact, privacy concerns seem to decrease if people have at least the impression that they are more in control of their data (Phelps et al., 2000; Smith et al., 2011). Additionally, privacy concerns, similar to creepiness, relate to people’s affective impressions about technologies. More precisely, if people are concerned about their privacy, it can induce uneasy feelings (Powell, 2013). Therefore, we propose:

Hypothesis 1a: Creepiness is positively correlated to privacy concerns.

Computer anxiety can be defined as an uncomfortable feeling when interacting with a computer or when there is the possibility that one has to use a computer (Chua et al., 1999; Barbeite and Weiss, 2004). Accordingly, creepiness relates to computer anxiety as people who are generally more anxious when it comes to interacting with a computer might also be people who will experience higher levels of creepiness when it comes to technology-related situations. Thus, we propose:

Hypothesis 1b: Creepiness is positively correlated to computer anxiety.

Transparency of a situation is given if people understand what is going on during this situation (Truxillo et al., 2009; McCarthy et al., 2017). In contrast, if people conceive that there is something shady about the situation or that they do not see through a situation, this reduces transparency. It is likely that situations that are not transparent are also creepy because if a situation is not instantly clear, people might come up with several (possibly wrong) explanations about this situation, thus increasing ambiguity (see also Studies 1 and 2). For instance, in the case of personalized advertising for baby products, people might start to wonder how the providers of these advertisements know about a woman’s pregnancy. Conversely, if the providers of the advertisement made clear from where they received their information, this situation would be less ambiguous, more predictable, and thus less creepy. We therefore propose:

Hypothesis 1c: Creepiness is negatively correlated to transparency. This relation might be more pronounced for creepy ambiguity.

The more people perceive that they are able to influence a situation, the more they think it is controllable (Ajzen, 2002). If a person’s behavior makes no difference regarding the outcome of a situation, the situation is uncontrollable, possibly leading to negative feelings about the situation and everything associated with it (Venkatesh et al., 2003). For example, people trying to avoid personalized advertising might be successful so long as their friends and family do not spend time on the internet. When a friend allows apps to access contact information on their smartphones, advertisement can become personalized for the person who originally tried to avoid it (Shklovski et al., 2014). Consequentially, these people no longer feel in control of personalized advertising because no matter what they do, advertisers will be able to obtain information about them that they will use to personalize advertisements. This lack of control might also lead to unpredictability, as it is less possible to influence the future within uncontrollable situations. As such, perceived control also relates to creepiness as decreased predictability increases the creepiness of situations (Tene and Polonetsky, 2015; McAndrew and Koehnke, 2016). Furthermore, low controllability might especially be related to emotional aspects of creepiness, as low controllability seems to relate to negative affective impressions (cf., Tamir et al., 2007). We thus propose:

Hypothesis 1d: Creepiness is negatively correlated to controllability. This relation might be more pronounced for emotional creepiness.

### Divergent Validity

To provide evidence for divergent validity, we chose the personality dimensions extraversion and conscientiousness, as both are expected to be unrelated to creepiness. In the case of extraversion, it should not matter if a person is especially outgoing or rather reserved in judging the creepiness of a situation. In the case of conscientiousness, a person who is rather lazy should be equally influenced by a creepy situation like a person who closely keeps track of their daily schedule. Therefore, we propose:

Hypothesis 1e: Creepiness is not (or at least to a lower extent in comparison to the convergent validities) correlated to extraversion.

Hypothesis 1f: Creepiness is not (or at least to a lower extent in comparison to the convergent validities) correlated to conscientiousness.

### Study 3: Method

We used G^∗^Power (Faul et al., 2009) to calculate that *N* = 153 participants are necessary for an assumed correlation of *r* = 0.20 and a power of 1-β = 0.80. Three participants were excluded because they stated that their data should not be used for the analysis, one participant was excluded because of very fast response times to the items (e.g., taking only 2 s for four items), and one further participant was excluded because of staying on the page on which the video was shown for nearly 15 min, indicating that s/he did not pay attention to the video. Participants were recruited via social networks and an online survey platform on which researchers take part in online surveys in exchange for other people to take part in their surveys. The final sample consisted of 153 German participants (73% female) with a mean age of 23.61 years (*SD* = 12.13) and a range of 18–60 years. Participants were predominantly students (84%). Most of them studied psychology (62%), and 12 percent of the participants studied business. Additionally, more than half of the participants (51%) indicated that they were currently working (65% of these part-time, the rest on average 45 h per week).

The study was conducted via an online survey platform and participants watched the same video as in the first and second study. Thus, participants also evaluated the items in regard to an interaction with a technology which potentially evokes creepiness. Afterward, they responded to the CRoSS, the other measures assessing convergent and divergent validity, demographic questions, and (similar to Study 1 and 2) to an open-ended question in which they were required to describe what has happened during the situation in the video.

### Study 3: Measures

All measures except for extraversion and conscientiousness were rated on a scale from 1 (*strongly disagree*) to 7 (*strongly agree*).

Privacy concerns were measured with six items adapted to the purpose of this study; taken from Langer et al. (2017, 2018).

Computer anxiety was measured using four items from Barbeite and Weiss’s (2004) scale. A sample item was: “Working with a computer would make me very nervous.”

Transparency was measured with three items. Two of these items were taken from Langer et al. (2018) and adapted to the purpose of this study, and we developed one additional item (“It was clear what was happening during the situation in the video.”)

The four controllability items were taken from Langer et al. (2017) who followed suggestions from Ajzen (2002). We adapted these items to the purpose of this study. A sample item was: “I am convinced that I could control the situation shown in the video.”

For conscientiousness and extraversion we used a German measure of the Big Five Inventory by Rammstedt and John (2005) with four items for each of the dimensions rated from 1 (*disagree strongly*) to 5 (*agree strongly*). A sample item for conscientiousness was: “I see myself as someone who does things efficiently.” A sample item for extraversion was: “I see myself as someone who is outgoing, social.”

### Study 3: Results

Table 5 shows a few examples participants provided on their explanations for the situation. Findings showed that they came up with similar explanations to participants in Studies 1 and 2.

**Table 5:** Explanation that participants came up with in Study 3.

Explanation type	Example
Creepy situation	–The computer froze and did not restart. During texting a friend (but before sending the message) a supposed customer support called.
	–Computer crashed. Person reacts hectically, searches for help and contacts a fried. Receives a call from an employee of the technical support within her company. She is obviously being monitored.
Hacker attack	–Somebody was hacked and is supposed to provide her data and pay money.
	–During the use of a chat-program the data were submitted to someone else.
Description of the situation	–Writing a document – computer did not respond any more – texting a friend for help – instantly called by the computer service that offered help.
	–A person worked at the computer as the mouse suddenly stopped working. Afterward, the person shut down the computer and texted someone for help. Then the person received a call offering solutions.

*These explanations were translated from German.*

Table 6 presents correlations and reliabilities of the study variables. Regarding convergent validity, Hypotheses 1a–d were all supported as the results showed significant correlations between the creepiness scale and privacy concerns, computer anxiety, transparency, and controllability. As hypothesized, privacy concerns and computer anxiety were positively correlated, whereas controllability and transparency were negatively correlated with creepiness. Furthermore, we found additional support for Hypothesis 1c as the results showed that transparency only correlated with creepy ambiguity, whereas there was no significant correlation between transparency and emotional creepiness.

**Table 6:** Correlations between the study variables of Study 3.

	Scale	*M* (*SD*)	1	2	3	4	5	6	7	8	9	10	11
1	Emotional Creepiness	4.77 (1.21)	0.82										
2	Creepy Ambiguity	4.54 (1.25)	0.59^**^	0.78									
3	Creepiness	4.66 (1.09)	0.89^**^	0.89^**^	0.86								
4	Age	23.61 (12.13)	0.03	-0.11	-0.05	–							
5	Gender	–	-0.30^**^	-0.27^**^	-0.32^**^	0.01	–						
6	Privacy Concerns	5.55 (1.01)	0.34^**^	0.29^**^	0.36^**^	-0.04	-0.08	0.86					
7	Transparency	4.13 (1.40)	-0.14	-0.34^**^	-0.27^**^	-0.05	0.12	0.09	0.81				
8	Controllability	3.45 (1.15)	-0.35^**^	-0.34^**^	-0.39^**^	0.03	0.21^**^	-0.24^**^	0.22^**^	0.81			
9	Computer Anxiety	2.28 (1.20)	0.25^**^	0.24^**^	0.27^**^	-0.13	-0.26^**^	0.08	-0.12	-0.26^**^	0.88		
10	Conscientio-usness	3.85 (0.63)	-0.06	-0.04	-0.06	0.07	0.02	-0.07	-0.06	-0.03	-0.02	0.70	
11	Extraversion	3.55 (0.98)	-0.07	0.02	-0.02	-0.08	-0.08	-0.14	-0.12	0.05	0.04	0.19^*^	0.90


*Coding of Gender: 1 = female, 2 = male. The numbers in the diagonal represent Cronbach’s alpha of the scales. *N* = 153.*

*^∗^*p* < 0.05, ^∗∗^*p* < 0.01.*

In contrast to the second part of Hypothesis 1c, there was no difference in the magnitude of correlations between the subdimensions of creepiness and controllability.

Regarding divergent validity, the results (cf. Table 6) showed support for Hypotheses 1e and 1f. Neither the entire creepiness scale, nor its subdimensions correlated significantly with extraversion and conscientiousness.

In a last explorative step, we assessed the relations between creepiness and participants’ gender and age. The results showed that females expressed higher feelings of creepiness compared to male participants, and that there was no significant relation between creepiness and participants’ age.

To summarize, the results of Study 3 increased our understanding of the construct of creepiness and its nomological network. Study 3 showed that creepiness is positively related to computer anxiety and privacy concerns, negatively related to transparency (especially creepy ambiguity) and controllability, whereas it is not related to conscientiousness, extraversion, or participants’ age. Taken together, these results provide support for the convergent and divergent validity of the CRoSS and its subscales. Lastly, Study 3 showed initial support of the assumption by former research that females might express higher feelings of creepiness than males (McAndrew and Koehnke, 2016). In the experimental design of Study 4, this finding will be investigated more closely, together with the assumption that creepiness is a feeling that can also be expressed in real-life situations.

## Study 4: Validation in a Real-Life Situation

In this last step of our scale development, we applied the CRoSS to a real life-situation. Throughout the previous three studies, participants only watched a video involving a creepy situation with a technology. However, creepiness should also be present in situations that do not use technology. Therefore, in Study 4, participants were either approached by a male or a female experimenter in a public place where they were asked to respond to the CRoSS items. This was either done during the day, or at night.

McAndrew and Koehnke (2016) proposed that men will be evaluated as being creepier than women. A reason for this could be that males are, in general, more physically threatening and underlie the stereotype of being more violent than women (McAndrew and Koehnke, 2016). On the one hand, this could mean that people are more afraid of men. On the other hand, this also implies that men are perceived as being less predictable and potentially less controllable than women, so other males and females might be constantly aware of a possible threat by males.

Additionally, McAndrew and Koehnke (2016) proposed that women in general feel more creepiness in most situations. This might be true because “being weak” is a common stereotype for females (Eagly and Steffen, 1984; Rosette and Tost, 2010). People who think they are weak might also think that they are less able to control a variety of situations. Therefore, we propose:

Hypothesis 2a: A male experimenter will evoke more creepiness than a female experimenter.

Hypothesis 2b: Women will report more creepiness than men.

Furthermore, environmental aspects can also evoke creepiness. For instance, McAndrew and Koehnke (2016) describe a dark tunnel as an example of a creepy environment. In addition, Watt et al. (2017) stated that people are more likely to come across creepy people at night, and Boomsma and Steg (2014) proposed that people feel more queasy at night. The night relates to our concept of creepiness such that at night people might have the feeling that they are less able to predict what will happen, and that situations that occur at night are less transparent, simply because people cannot perceive their surroundings as well as during the day. Thus, we propose,

Hypothesis 2c: The experimental situation during the night will evoke more creepiness than during the day.

### Study 4: Method

For the fourth study we calculated the required sample size using G^∗^Power (Faul et al., 2009). For a power of 1-β = 0.80 and a moderate effect size for the interaction effect, a sample size of 128 participants was required. Therefore, we collected data from 128 participants (53% female) with an average age of 34 years (*SD* = 12.09), ranging from 18 to 69 years.

**FIGURE 2:**
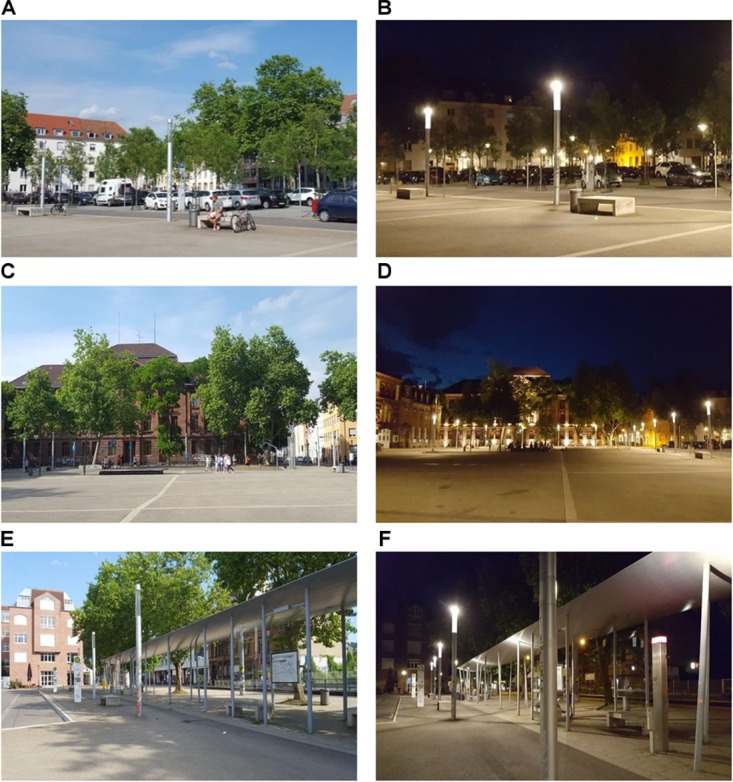
**(A–F)** Pictures of the public place where participants were contacted during the day or at night. Copyright Josephine Malsch.

In a 2 × 2 × 2 design (male experimenter vs. female experimenter; male participant vs. female participant; day vs. night) we chose a public place to contact participants (see Figures 2A–F). Our experimenters received a script instructing them to dress similarly, to not smile at participants, and to not behave especially friendly, but still politely. In addition, they were told to never collect data at the same time as the other experimenter. The experimenters were both Caucasian, had blue eyes and bright skin, and were 26 years old. The female experimenter was 171 cm tall (5′6″) and the male experimenter was 174 cm (5′7″). In 2 weeks in May (only on weekdays, and only on days/nights when it was not raining), the experimenters went to the public place and approached people to fill out the CRoSS items. Participants were instructed to rate the situation they had just experienced (i.e., the situation of being contacted by a stranger to fill out a questionnaire). The hours of data collection during the day were between 3 pm and 6 pm, for data collection that took place at night, the hours were from 10 pm until 12 pm.

### Study 4: Results

Table 7 presents correlations between the study variables. Reliability for Creepiness was Cronbach’s α = 0.92 (emotional creepiness Cronbach’s α = 0.89; creepy ambiguity Cronbach’s α = 0.86).

**Table 7:** Correlations between the study variables of Study 4.

	Scale	*M* (*SD*)	1	2	3	4	5	6	7
1	Emotional Creepiness	2.25 (1.14)							
2	Creepy Ambiguity	3.00 (1.29)	0.73^**^						
3	Creepiness	2.62 (1.13)	0.92^**^	0.94^**^					
4	Participants’ Age	33.52 (12.09)	0.06	0.01	0.04				
5	Participants’ Gender	–	0.33^**^	0.27^**^	0.32^**^	0.00			
6	Experimenters’ Gender	–	0.07	0.19^*^	0.14	-0.20^*^	-0.03		
7	Time of the Day	–	0.15	0.27^**^	0.23^**^	-0.14	0.06	0.00	


*Coding of participants’ and experimenters’ gender: 1 = male, 2 = female, coding of time of the day: 1 = day, 2 = night. *N* = 128.*

*^∗^*p* < 0.05, ^∗∗^*p* < 0.01.*

To evaluate Hypotheses 2a–d we used an ANOVA with three factors. Means and standard deviations of the groups are presented in Table 8. In Hypothesis 2a it was assumed that a male experimenter will evoke more creepiness than a female experimenter. However, we found that the female experimenter evoked more creepiness, *F*(1,120) = 4.16, *p* < 0.05, $\eta_{p}^{2}$ = 0.03. Thus Hypothesis 1 was not supported.

**Table 8:** Means and standard deviations for the combinations of the independent variables gender of the experimenter, gender of the participant, and time of the day.

	Male experimenter				Female experimenter			
		
	Male participant		Female participant		Male participant		Female participant	
				
Group	Day *M* (*SD*)	Night *M* (*SD*)	Day *M* (*SD*)	Night *M* (*SD*)	Day *M* (*SD*)	Night *M* (*SD*)	Day *M* (*SD*)	Night *M* (*SD*)
Emotional Creepiness	1.65 (0.77)	1.67 (0.93)	2.13 (1.11)	2.94 (1.21)	2.40 (1.22)	2.83 (1.15)	2.13 (1.00)	1.91 (1.05)
Creepy Ambiguity	1.93 (0.86)	2.60 (0.94)	2.59 (1.21)	3.69 (1.01)	3.13 (1.40)	3.75 (1.31)	2.99 (1.31)	3.08 (1.32)
Creepiness	1.79 (0.69)	2.13 (0.79)	2.36 (1.09)	3.32 (1.02)	2.77 (1.23)	3.29 (1.16)	2.56 (1.09)	2,49 (1.07)
*n*	17	12	15	20	17	16	15	16


*N = 128.*

Furthermore, we found support for Hypothesis 2b, as women reported more creepiness than men, *F*(1,120) = 13.81, *p* < 0.01, $\eta_{p}^{2}$ = 0.10. In addition, Hypothesis 2c was supported as participants who were approached at night expressed more creepiness than participants who were approached during the day, *F*(1,120) = 5.63, *p* < 0.05, $\eta_{p}^{2}$ = 0.05.

## Discussion

The current paper introduced the Creepiness of Situation Scale as a measure to examine creepiness of various situations. Following rigorous psychometrical guidelines for scale development by Hinkin (1998), the four current studies show that the CRoSS offers a reliable measure of general creepiness and its two subdimensions emotional creepiness and creepy ambiguity. It therefore offers an additional perspective to evaluate novel technologies over and above scales based on the TAM (Venkatesh et al., 2003), on usability aspects (Laugwitz et al., 2008), and on eeriness (Ho and MacDorman, 2010). Furthermore, the CRoSS could be a valuable tool to advance research on creepiness in interpersonal situations. Study 1, which used an American sample, showed that the CRoSS consists of two subdimensions. Study 2 confirmed these two correlated subdimensions of creepiness in a German sample. Finally, Studies 3 and 4 supported the validity of the CRoSS in a technological and in an interpersonal real-world context. Additionally, the results from Study 4 indicated that the CRoSS is sensitive to experimental manipulations based on theoretical assumptions.

As we explained in the introduction, organizations nowadays constantly come up with new services in which algorithms judge human behavior (e.g., personalized advertising, Shklovski et al., 2014), people are repeatedly exposed to novel technological inventions (e.g., self-driving cars, Tene and Polonetsky, 2015), and humans increasingly interact with virtual characters and robots (Langer et al., 2018). One word to describe feelings of uncertainty about how to feel during these situations and how to judge these situations seems to be “creepy.” Previous research has tried to define the term creepiness (e.g., Tene and Polonetsky, 2015; McAndrew and Koehnke, 2016), and has measured creepiness with single-item measures (e.g., Inkpen and Sedlins, 2011; Watt et al., 2017). However, no study so far has attempted to integrate theoretical assumptions regarding creepiness to develop a sound measure for creepiness.

One shortcoming of previous creepiness measures is that they were not developed to fulfill basic psychometrical standards. For creepiness research to evolve however, and to make results from different studies on creepiness comparable, there is need for a psychometrically sound measure of creepiness. For single-item measures, it is not possible to provide information about Cronbach’s Alpha reliability values, whereas the CRoSS shows good to very good Cronbach’s Alpha values throughout all four current studies. For measures lacking theoretical background, it is hard to come up with theoretical assumptions about its relation to other important measures. It is even harder to develop specific hypotheses. This might be a reason why research has yet to provide validity data on the relations between creepiness and other measures. Regarding validity of the CRoSS, it was possible to generate theory-based hypotheses concerning the relation of creepiness with other relevant measures and to predict the direction of these relations (e.g., a positive correlation with transparency, but only for creepy ambiguity). All in all, our results regarding reliability and validity suggest that the CRoSS is a potentially useful scale to advance research on creepiness.

An additional contribution of the current set of studies is that the findings suggest that creepiness can be differentiated into two subdimensions, creepy ambiguity and emotional creepiness. This differentiation can help to increase our understanding of the creepiness concept. One example for this increased understanding can be found in Study 3 that found that non-transparent situations evoke creepy ambiguity, but to a lesser extent emotional creepiness. This indicates that increasing transparency may help to decrease creepy ambiguity. In contrast, influencing situations which involve the affective dimension of creepiness might require other interventions. For example, it is imaginable that, similar to other negative emotional impressions (e.g., eeriness, anxiety; MacDorman, 2006; Powers and Emmelkamp, 2008), emotional creepiness also declines the more a situation becomes familiar. These insights would not have been possible with a single-item measure of creepiness.

### Theoretical Implications

Speaking in favor of the value of the CRoSS for research on creepiness, the current studies support and extend previous research regarding creepiness (e.g., Tene and Polonetsky, 2015; McAndrew and Koehnke, 2016). Our studies show that creepiness relates to variables that are associated with the predictability of a situation (i.e., less transparency and controllability). This enhances our understanding of the creepiness concept as the results provide insight into creepiness’ nomological network.

Study 4 also lends further support for the relation of creepiness and predictability. During the day, it might be more common to interact with people who contact you to fill out some questionnaires, whereas an experimenter who approaches people at night to fill out a questionnaires is rather uncommon. Therefore, participants who realized that an experimenter is approaching them during the night had a harder time predicting what will happen next than participants exposed to the same situation during the day.

Furthermore, our findings support assumptions of Shklovski et al. (2014) who proposed that privacy concerns are related to creepiness. Studies 1–3 exposed participants to a situation that was interpreted as evoking privacy concerns. Participants concluded that the customer support was acting like “Big Brother” (see Table 3), or that the situation was a “disturbing breach of privacy” (see Table 2). At the same time, Study 3 found that participants who perceived the situation as a more severe instance of privacy invasion also reported higher feelings of creepiness.

Another field of research that could benefit from the CRoSS is research regarding the uncanny valley. As stated in the theoretical background, this field of research has produced mixed results (cf., Kätsyri et al., 2015). We posit that the CRoSS might be a useful tool to explore the uncanny valley in a more standardized fashion. Future studies regarding the uncanny valley may use the CRoSS as an additional and broadly applicable way of measuring its impact, thus making results more comparable. It would be especially interesting to investigate the relation of creepiness and eeriness (Ho and MacDorman, 2010) in research regarding the uncanny valley. Up to now, we can only speculate that emotional creepiness will be especially related to eeriness. Furthermore, the CRoSS might be of particular value when investigating the uncanny valley during interactions with robots and virtual characters where there is still only scarce research, because previous work on the uncanny valley has predominantly used the evaluation of pictures or videos depicting robots or virtual characters (Kätsyri et al., 2015).

Aside from its usefulness within technological settings, the CRoSS also seems to be a valuable measure for assessing creepiness in other real-life situations. For instance, we found support for assumptions by McAndrew and Koehnke (2016) that women in general express higher feelings of creepiness. This result is similar to findings from previous research which has shown that women tend to report more pronounced affective reactions than men (Ashmore, 1990; Ho et al., 2008). This supports the assumption that creepiness has an affective component. In contrast, our results question the expectation of McAndrew and Koehnke (2016) that men evoke higher feelings of creepiness. The results from Study 4 indicate that a female experimenter induced more creepiness, implicating that women possess characteristics (e.g., body language, facial expressions, behavior) that are equally or even more likely to induce creepiness compared to physical threat evoked by men (cf., McAndrews and Koehnke, 2016). One possible explanation for this result is that we told our experimenters to be polite, but not to smile. Since females tend to be more emotionally expressive (Kring and Gordon, 1998), it might have been more unfamiliar for participants to be contacted by a female experimenter who did not smile as opposed to a non-smiling male experimenter, thus leading to higher feelings of creepiness. If the reason for more creepiness was unfamiliarity of the situation, this would again speak in favor of the assumption that predictability is related to creepiness. More precisely, people in unfamiliar situations possess less knowledge about the situation and therefore they might be less able to predict what will happen next (cf., Eagly and Steffen, 1984). Therefore, we propose that creepiness might be elicited by situations challenging existing cognitive schemata thus reducing familiarity (Rumelhart, 1980). For instance, people might possess knowledge and experience about interacting with computers (e.g., they know it is possible to interact within virtual worlds using a mouse and a keyboard), and they therefore possess a schema about “computer interaction.” If their new computer suddenly is able to recognize non-verbal behavior (e.g., it is possible to interact within virtual worlds using voice and smiles), an unfamiliar new aspect is added to the familiar interaction with the computer which could elicit creepiness. However, it is important to note that the current study only showed initial support that creepiness relates to concepts associated with predictability and familiarity (e.g., transparency, controllability). The exact paths and causal relations between these concepts and creepiness need to be addressed in future experiments.

### Practical Implications

If researchers evaluate novel technologies, they might consider using the CRoSS as an additional evaluation criterion. Above and beyond scales based on the TAM and on usability aspects of technologies, the CRoSS offers an efficient and valid way of assessing participants’ affective reactions toward technology-enhanced situations. Previously, evaluations of technologies might have missed these aspects, as feelings of creepiness were not included in previous scales developed to assess user reactions (see Venkatesh et al., 2003). In fact, a study by Langer et al. (2017) speaks in favor of the validity of the CRoSS for technological settings. They used the CRoSS and showed that a novel technology-mediated job interview approach led to higher feelings of creepiness and that creepiness correlated negatively with important applicant reaction variables (e.g., organizational attractiveness). Additionally, another study by Langer et al. (2018) used the CRoSS to evaluate a job interview with a virtual character as the interviewer. Within their study, creepiness again correlated with relevant applicant reaction variables privacy concerns and transparency, thus replicating the findings of our validation approaches within Study 3 and speaking in favor of the usefulness of the CRoSS.

Furthermore, companies could use the CRoSS to improve acceptance of new products and services. For instance, organizations providing personalized advertising can investigate the creepiness of their services and try to decrease it. As our results show that transparency can diminish creepiness, it might be a promising way to provide information about how personalized advertisement is generated to reduce creepiness (see also McCarthy et al., 2017).

In addition, organizations producing robots or virtual characters (e.g., within movies) might be able to assess if their product is at risk of descending into the uncanny valley. For example, movie producing companies could show their virtual characters to a test audience, adapt and use the CRoSS as well as the eeriness index by Ho and MacDorman (2010), and compare different versions of their virtual characters regarding creepiness and eeriness. This way, undesirable surprises at the launch of the movie could be prevented.

### Limitations

There are at least three limitations that need to be addressed. First, Study 1 was conducted in an American sample, whereas the other three studies were conducted with German participants. Therefore, implications of Studies 2–4 regarding reliability and validity of the CRoSS might not be generalizable to the English version of the scale. However, comparing Study 1 and the other studies shows that the two-factor solution that was found in the American sample generalized to the German samples, that Cronbach’s α of the scales was similar for all of the studies, and that there were no differences between the countries regarding the level of creepiness induced by the experimental video. This initially supports that the German and English version of the CRoSS are comparable.

Second, Studies 1–3 were all conducted online and participants only watched a video instead of interacting directly within a creepy situation. A consequence of this could be that results would have been different if people had interacted directly within the situation. However, Study 4 supports the assumption that the CRoSS also works to evaluate creepiness in real-life situations.

Third, we did not directly measure basic emotions that potentially relate to creepiness. For instance, we posited in the theory that creepiness should be correlated to anxiety and found that creepiness corresponded to computer anxiety. However, we did not measure anxiety as a state during the respective situations (e.g., whilst being approached by a stranger at night). Following Ho et al. (2008), emotions such as disgust and fear could also be relevant in the realm of creepiness. Therefore, future studies on creepiness should consider measuring emotional states during interactions and situations in order to clarify the relation of creepiness to other emotions.

### Future Research

Future research should aim to examine the predictive validity of the CRoSS. For instance, it would be interesting to examine the mismatch hypothesis (i.e., the hypothesis that a mismatch between the human-like look of a virtual character or robot and its potentially artificial behavior is one reason for the uncanny valley; Kätsyri et al., 2015) using the CRoSS. Participants could interact with virtual characters or robots, which are experimentally manipulated regarding different levels of mismatch, and the CRoSS can be used to assess if increases in mismatch also increases creepiness.

Additionally, the CRoSS is likely to be an important measure for research into trust in automation (Hoff and Bashir, 2015). Trust in automation is concerned about antecedents and consequences of trust when humans interact with automated systems (Hoff and Bashir, 2015). Trust is required in uncertain situations (i.e., because it is unclear if a system will fulfill its job adequately), and affective impressions seem to be especially important in the formation of trust (Lee and See, 2004). As we have argued in the theoretical background of this paper, uncertainty and affective impressions are two main aspects of creepiness in a situation, and creepiness might thus influence humans’ interactions with automated systems. For instance, an automated system might elicit creepiness because it is unclear to the human user what is happening during the situation, and users could therefore distrust the system.

Additionally, it could be a fruitful approach to experimentally manipulate the level of creepiness through reducing controllability and transparency of a technology. For instance, a virtual trainer providing feedback for non-verbal behavior (cf., Langer et al., 2016) might be less creepy if it provides participants with information about its functionality, and if it appears to be manageable and clear that participants can influence outcomes and feedback through their own behavior. Such studies could help to further enhance our understanding of the creepiness construct.

Additionally, the authors would like to stress that the CRoSS is not restricted to situations using novel technologies; rather its uses can be extended to other real-life situations that are supposed to elicit queasy feelings and ambiguity. For instance, the CRoSS could be used to evaluate the creepiness of a public parking deck. If participants report that they perceived creepiness when walking through the parking deck, installation of further illumination could help reduce those feelings.

Lastly, translating and validating the CRoSS in other languages might lead to intercultural comparability of the creepiness concept. It could be that there are cultures and countries whose people experience lower feelings of creepiness. For instance, people in countries scoring low on Hofstede’s dimension of uncertainty avoidance (cf., Hofstede, 1984) might be less sensitive to creepiness as they tend to be better at handling uncertainty, unpredictability, and ambiguity. Hence, it might be a fruitful direction for future research to investigate creepiness in different cultures.

## Conclusion

The current study increased our understanding of the creepiness concept. With the CRoSS, we developed and validated a scale that can advance research on creepiness. Moreover, the CRoSS might provide a new, formerly neglected, perspective on the evaluation of technologies in research and practice.

## Ethics Statement

This study was carried out in accordance with recommendations of the German Psychological Society that state that research like the one reported here is exempt from formal Ethics Committee approval. All subjects gave their informed consent in accordance with the Declaration of Helsinki.

## Author Contributions

ML was mainly responsible for the idea, conception of the studies, data collection, analysis, interpretation, and writing of the article. CJK was predominantly involved in conception of the studies, feedback on data interpretation, and writing of the article.

## Conflict of Interest Statement

The authors declare that the research was conducted in the absence of any commercial or financial relationships that could be construed as a potential conflict of interest.

## Footnotes

1 Following the call for open science (Open Science Collaboration, 2015) every study of this paper was pre-registered. This means, before we collected any data we pre-registered our hypotheses as well as the intended data collection and analysis approaches. Hyperlinks to the pre-registrations can be found in the acknowledgements.
